# Predictive utility of prior positive urine culture of extended- spectrum β -lactamase producing strains

**DOI:** 10.1371/journal.pone.0243741

**Published:** 2020-12-14

**Authors:** Basima A. Almomani, Rawand A. Khasawneh, Rola Saqan, Munther S. Alnajjar, Lara Al-Natour

**Affiliations:** 1 Department of Clinical Pharmacy, Faculty of Pharmacy, Jordan University of Science and Technology, Irbid, Jordan; 2 Department of Pediatrics and Neonatology, Faculty of Medicine, Jordan University of Science and Technology, Irbid, Jordan; 3 Department of Biopharmaceutics & Clinical Pharmacy, College of Pharmacy, Al-Ahliyya Amman University, Amman, Jordan; Medical University of Gdansk, POLAND

## Abstract

Rising incidence of extended- spectrum beta-lactamase (ESBL) induced urinary tract infections (UTIs) is an increasing concern worldwide. Thus, it is of paramount importance to investigate novel approaches that can facilitate the identification and guide empiric antibiotic therapy in such episodes. The study aimed to evaluate the usability of antecedent ESBL-positive urine culture to predict the pathogenic identity of future ones. Moreover, the study evaluated the accuracy of selected empiric therapy in index episodes. This was a retrospective study that included 693 cases with paired UTI episodes, linked to two separate hospital admissions within 12 month-period, and a conditional previous ESBL positive episode. Pertinent information was obtained by reviewing patients’ medical records and computerized laboratory results. Multivariate analysis showed that shorter interval between index and previous episodes was significantly associated with increased chance of ESBL-positive results in current culture (OR = 0.912, 95CI% = 0.863–0.963, p = 0.001). Additionally, cases with ESBL-positive results in current culture were more likely to have underlying urological/surgical condition (OR = 1.416, 95CI% = 1.018–1.969, p = 0.039). Investigations of the accuracy of current empirical therapy revealed that male patients were less accurately treated compared to female patients (OR = 0.528, 95CI% = 0.289–0.963, p = 0.037). Furthermore, surgical patients were treated less accurately compared to those treated in internal ward (OR = 0.451, 95CI% = 0.234–0.870, p = 0.018). Selecting an agent concordant with previous microbiologic data significantly increased the accuracy of ESBL-UTIs therapy (p<0.001). A quick survey of the previous ESBL urine culture results can guide practitioners in the selection of empiric therapy for the pending current culture and thus improve treatment accuracy.

## Introduction

Urinary tract infections (UTIs) are among the most common bacterial infections in adults; accounting for approximately 20–40% of infectious cases [1, 2]. *Escherichia coli* (*E*. *coli*), and *Klebsiella pneumoniae* (*K*. *pneumoniae*) are usually the most commonly implicated species [3]. *G-negative cocci*, *enterococci*, and *Staphylococcus saprophyticus* are also involved [4]. In recent years, extended- spectrum beta-lactamase (ESBL) producing strains have been reported with higher frequency worldwide [5, 6]. ESBL positivity signifies the production of enzymes that can destroy and hydrolyze most beta lactam antibiotics. Identification of ESBL positive isolates is quite alarming since it confers resistance to commonly used antibiotics, including penicillins, cephalosporins, and monobactams, rendering them ineffective in treating resultant UTIs [7].

Generally, UTIs are classified based on location of infection within urinary tract, the presence of risk factors, and the presence of associated symptoms. The risk of UTIs increases with age, except for the peak incidence noticed in younger females between 14–24 years of age [1]. General treatment approach in the context of potential UTIs include initiation of empiric antibiotics while the results of definite urine cultures are pending. General factors that are usually considered during the selection of any empiric regimen include: the site of infection, the most frequent causative pathogens, the place of acquisition (i.e. community or hospital-acquired), in addition to regional and institution-specific antimicrobial susceptibility patterns [8]. Carbapenems are the usual antibiotic class option for ESBL-induced UTIs; if local resistance pattern suggests high prevalence of ESBL-producing strains, ertapenem is among one of the recommended first line drugs [9]. However, observations of carbapenem-resistant infections have been reported [10]. Thus, the excessive use of these agents can speed up the emergence of carbapenem-resistant strains which further limits therapeutic options to aminoglycosides, polymyxins, tigecycline, fosfomycin, and temocillin which are all associated with considerable risk of toxicities, resistance potential, and descent efficacy [11].

Inappropriate empiric antibiotic selection in ESBL-induced infections has been associated with worse clinical outcomes, increased morbidity and mortality, enhanced development of antibiotic resistant strains, and increased risk of treatment failure [12, 13]. In this light, two perspectives are of concern to healthcare professionals when choosing empiric therapy [14]: firstly, judicious/vigilant approach to ESBL-induced infections that could lead to sub-optimal anti-infective treatment along with potential treatment failure, and increased morbidity and mortality. Secondly, anticipating diagnosis approach to potential ESBL infections that can lead to over-prescription of broad-spectrum empiric antibiotics; contributing to secondary infections and fueling the selection of resistant strains.

An increase in the prevalence of ESBL-producing isolates has been noticed in Jordanian hospitals [10, 15, 16]. The presence of preceding ESBL positivity in different types of infections, including UTIs, signify a potential colonization and can be employed to optimize empiric antibiotic therapy selection in future episodes [17–19]. The main purpose of this study was to evaluate the utility of a prior positive urine culture for ESBL producing isolates in predicting the results of a subsequent urine culture. The secondary aim was to evaluate the accuracy of empiric therapy as well as factors that might improve the accuracy of empiric antibiotic selection in patients with ESBL-UTIs.

## Materials and methods

### Ethics statement

The ethical approval to conduct this research was granted by the institutional review boards in Jordan University of Science and Technology (1/125/2019). Informed consent was waived for this retrospective study.

### Study setting

A retrospective study was conducted at King Abdullah University Hospital (KAUH), a 543-bed tertiary referral teaching hospital in north Jordan with an annual average admission rate of 40,350 patients.

### Case selection

All culture-confirmed UTI cases were reviewed from January 2014 to December 2019 from hospital database. All potentially eligible hospitalized adult and pediatric patients with at least one ESBL-positive UTI episode were identified, screened and accessed during the time period from September 2019 till March 2020. Patients diagnosed with at least two episodes of UTIs, linked to two separate hospital admissions, 12 months apart were recruited. The diagnosis of UTI was confirmed based on signs and symptoms in addition to a positive urine culture. Antecedent ESBL-UTI episode was the condition for inclusion. Cases with interval less than 14 days or more than 12 months between paired episodes or encounters were excluded.

The bacterial growth and sensitivity patterns in the index episode were compared to those retrieved from the previous admission. Identification of the causative pathogen and antibiotic susceptibility (current and previous) data were reported by the microbiology laboratory in the hospital (S1 File). Empirical therapy was considered concordant with previous culture if the patient received proper therapy as per guidelines and previous microbiological data. Accurate therapy was defined as the in vitro susceptibility of empiric therapy in ESBL-UTI.

To investigate predictors of urine culture pathogenic identity of the index episode, all episodes were divided into two groups: episodes with ESBL isolates and episodes with non-ESBL isolates. In order to evaluate the accuracy of the current empirical therapy, only episodes with ESBL positive isolates that were given empirical therapy in the index episode and have susceptibility data were included. The required information for each patient was obtained by reviewing patients’ medical records and computerized laboratory results. The following demographic and clinical parameters (S2 File) were measured and compared in different patients: age, gender, comorbid disease states, reasons of admission, presence of urological or surgical conditions, and type of empirical therapy.

### Microbiology identification

This section was based on the standard operating procedures aligned by KAUH microbiology laboratory unit. All urine samples were inoculated into blood agar and MacConkey agar and incubated at 37°C for 24 hours. Isolates were primarily identified by morphological characteristics, pigment production, and gram-staining. Further verification of suspected strains (*E*. *coli* and *K*. *pneumonia*e) was conducted by confirmation of motility and other relevant biochemical tests.

Subsequent identification and confirmation of ESBL production (both manual and automated using VITEK2 system) were performed according to Clinical and Laboratory Standards Institute (CLSI) guidelines 2014 [20]. Phenotypic surveillance of ESBL production was carried out using the standard disk diffusion method, where more than one antibiotic was used to enhance the accuracy of ESBL detection. This was accomplished through the use of ceftazidime and cefotaxime alone and in combination with clavulanic acid; an increase of inhibition zone diameter (≥ 5 mm) around combination disk versus single disk confirmed ESBL production. Afterwards, confirmation of suspected ESBLs producers was done by using the combination disc method and double-disk synergy (DDS) method on Mueller–Hinton agar. Antimicrobial susceptibility testing was performed using Kirby–Bauer disc diffusion technique on Mueller–Hinton agar, as recommended by the CLSI guidelines 2014 [20].

### Statistical analysis

Continuous variables were presented as median [IQR], while categorical variables were presented as numbers and percentages. Univariate analysis was conducted using the Mann-Whitney test for continuous variables and Chi-square (χ2) test for categorical variables. In order to determine factors (predictors) that were independently associated with urine culture identity results and accuracy of empiric therapy, multivariate analysis using binary logistic regression was performed including all variables with p<0.25 on univariate analysis. Odds ratios (OR) and their 95% confidence intervals (95% CI) were calculated. Statistical significance was set at p value less than 0.05. Statistical Package for Social Sciences (SPSS Inc., Chicago, IL) version 23 was used.

## Results

**Fig 1:**
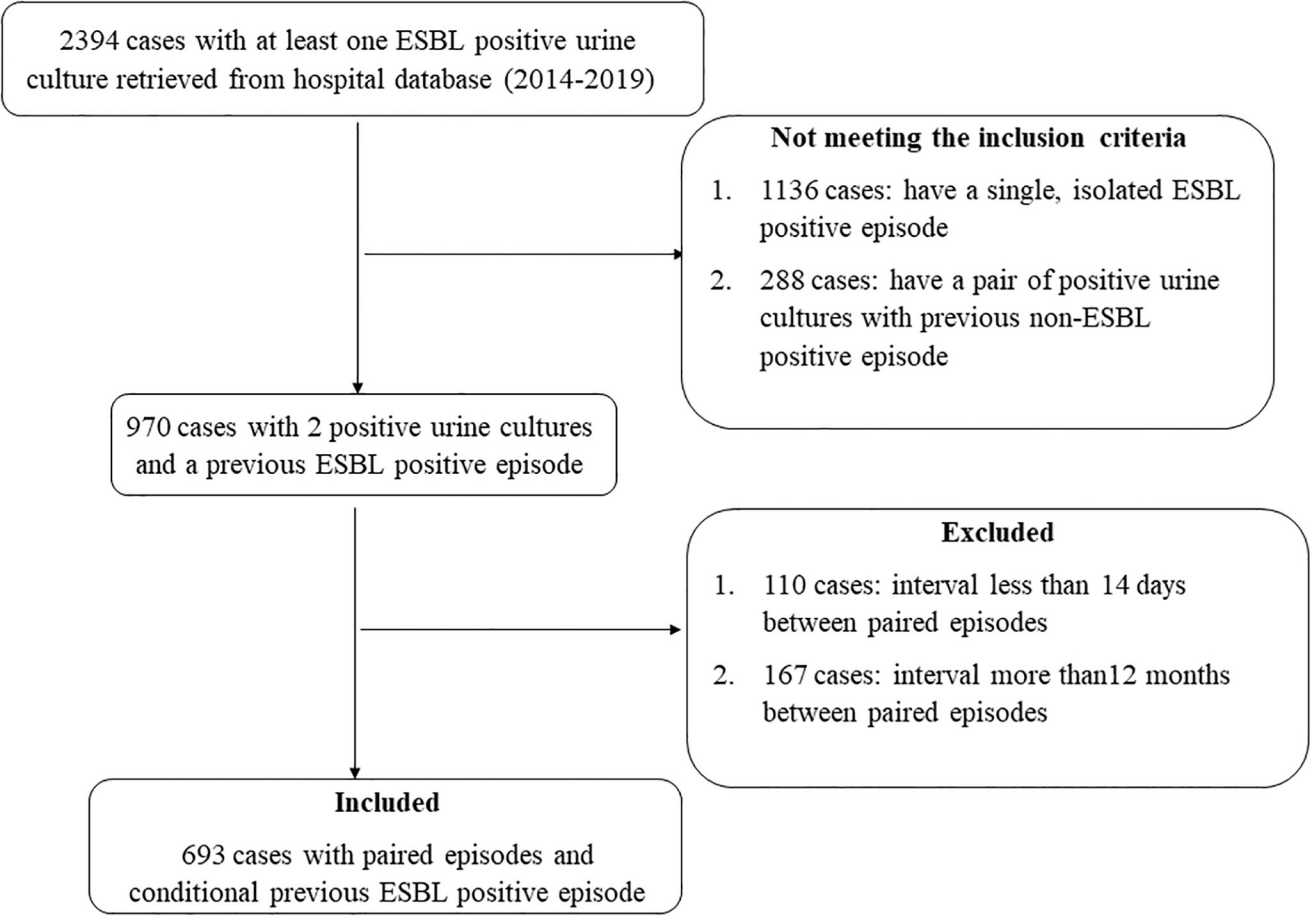
Summary of cases recruitment.

Out of the 2394 screened eligible hospitalized patients with at least one ESBL-positive UTI episode, 1424 cases were excluded since they failed to meet the inclusion criteria. During the study period, a total of 970 cases with paired urine cultures; and a conditional initial ESBL-positive UTI episode were identified from hospital database. Of those, 277 cases were excluded because there was either less than 14 days (110/277) or more than 12 months (167/277) between the two episodes, leaving 693 cases that were included in the final analysis. A detailed summary of the identification and inclusion process of cases is presented in Fig 1. The included 693 paired urine cultures were retrieved from 483 unique patients. The average age of patients was approximately 50 years and more than half of them were females (57.4%). Most cases were admitted to the hospital due to medical reasons (86.9%) and had a concomitant comorbid condition (78.4%). The median interval between paired isolates was 3 months. *E*. *coli* was the most common causative uropathogen in previous culture (82.3%) and the results of more than half of index urine cultures were ESBL-positive (58.6%). Importantly, 61% of the empirical therapy was concordant with prior microbiological data. A detailed description of demographic and clinical data is presented in Table 1.

**Table 1:** Demographic and clinical characteristics.

Characteristics^a^	All cases
Pairs of episodes	693
Number of patients	483
Years of recruited cases	
• 2014	65 (9.4)
• 2015	103 (14.9)
• 2016	149 (21.5)
• 2017	127 (18.3)
• 2018	99 (14.3)
• 2019	150 (21.6)
Age (years)^b^	59 [28–71]
Gender	
• Female	401 (57.4)
• Male	292 (42.1)
Comorbidty	
• No	150 (21.6)
• Yes	543 (78.4)
Interval between paired isolates (months)^b^	3 [2–5]
Reasons of admission	
• Medical reasons	602 (86.9)
• Surgical reasons	91 (13.1)
Presence of urological/surgical condition	
• No	390 (56.3)
• Yes	303 (43.7)
Type of previous microorganism	
• *E*. *coli*	570 (82.3)
• *K*. *pneumoniae*	123 (17.7)
Results of current urine cultures	
• ESBL-positive	406 (58.6)
• No growth	83 (12.0)
• Mixed growth	110 (15.9)
• Non-ESBL producers	86 (12.4)
• Fungi	8 (1.1)
Outpatients cultures	
• No	555 (80.1)
• Yes	138 (19.9)
Medication allergy	
• No	664 (95.8)
• Yes	29 (4.2)
Concordance of empirical therapy	
• Non-concordant	270 (39)
• Concordant	423 (61)

^a^ All data expressed as n (%) of participants unless otherwise indicated.

^b^ Data described as median [Interquartile range].

**Fig 2:**
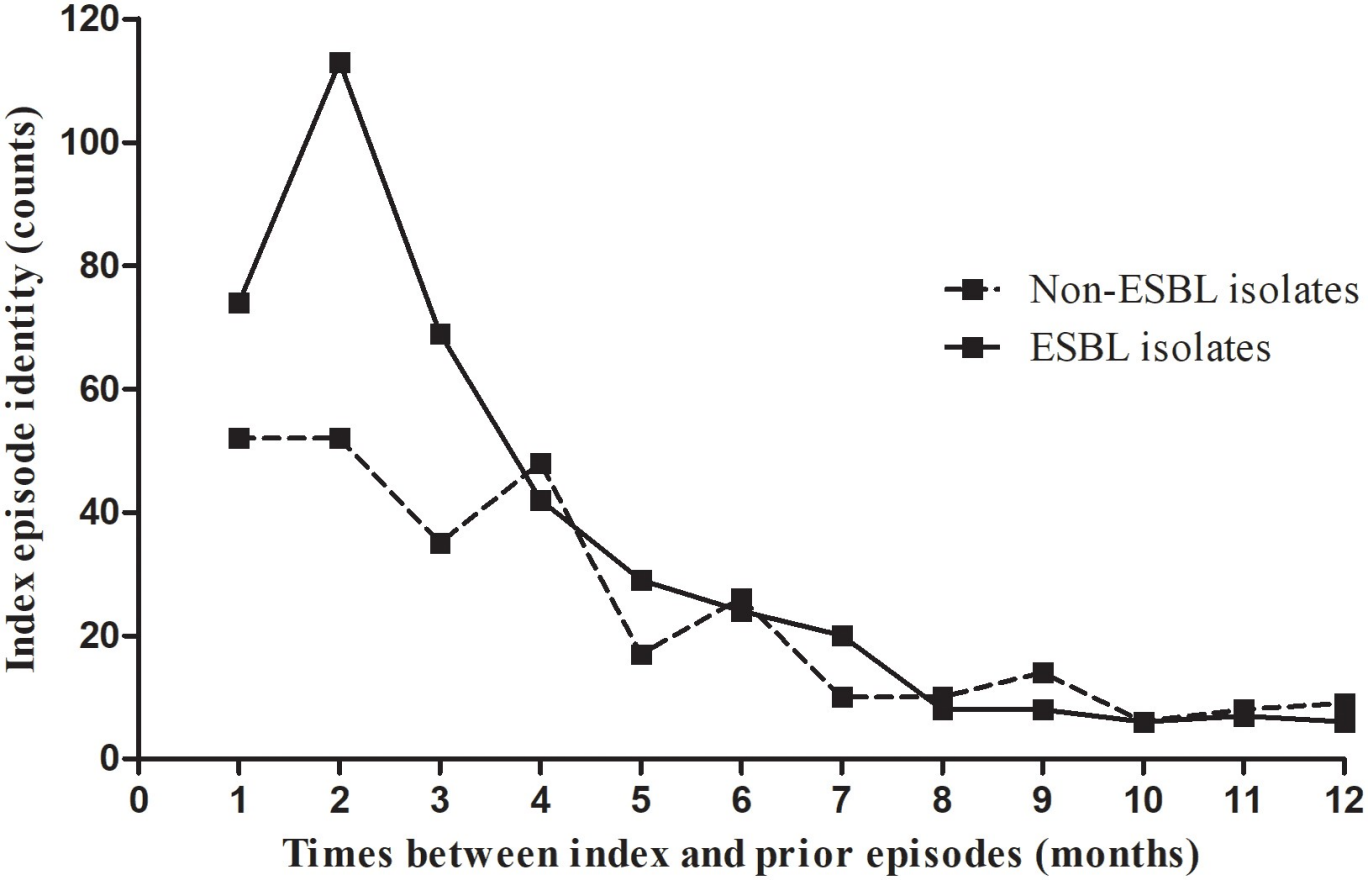
Relationship between monthly interval and index episode identity.

As shown in Table 2, the results of univariate analysis showed that gender (p = 0.020), presence of urological/surgical condition (p = 0.007) and interval between index and prior episodes (p = 0.003) were significantly associated with ESBL-positive result in the index episode. The multivariate analysis identified both presence of urological/surgical condition and interval between index and prior episodes as independent factors that predicted ESBL-positive result in the index culture. Cases with ESBL-positive result in the current culture were more likely to suffer from urological/surgical condition (OR = 1.416, 95CI% = 1.018–1.969, p = 0.039) (Table 2). In addition, shorter duration between index and prior episodes was significantly associated with increased chance of ESBL-positive result in the current culture (OR = 0.912, 95CI% = 0.863–0.963, p = 0.001) (Table 2 and Fig 2).

**Table 2:** Predictors of urine culture identity results of the index episode.

Factor^a^	Univariate analysis			Multivariate analysis		
	Non ESBL isolates N = 287	ESBL positive isolates N = 406	P value	OR	95%CI	P value
Age (years)^b^	60 [24–71]	58 [29.75–70]	0.949			
Gender			0.020			0.140
• Female	181 (63.1)	220 (54.2)		Ref.		
• Male	106 (36.9)	186 (45.8)		1.281	0.922–1.779	
Comorbidity			0.559			
• No	59 (20.6)	91 (22.4)				
• Yes	228 (79.4)	315 (77.6)				
Interval between paired isolates (months)^b^	4 [2–6]	3 [2–5]	0.003	0.912	0.863–0.963	0.001
Reasons of admission			0.194			0.512
• Medical reasons	255 (88.9)	347 (85.5)		Ref		
• Surgical reasons	32 (11.1)	59 (14.5)		1.173	0.728–1.892	
Presence of urological/surgical condition			0.007			0.039
• No	179 (62.4)	211 (52)		Ref.		
• Yes	108 (37.6)	195 (48)		1.416	1.018–1.969	

Abbreviations: OR, odds ratio; CI, confidence interval.

^a^ All data expressed as n (%) of participants unless otherwise indicated.

^b^ Data described as median [Interquartile range].

To investigate the accuracy of the current empirical therapy, we included cases with ESBL-positive culture whom received empirical antibiotic therapy in the analysis. Out of 406 episodes with current ESBL-positive culture, 121 cases were excluded due to one of either two reasons: (i) 4 cases did not have susceptibility data for the given empirical therapy and (ii) 117 cases were admitted to the hospital and given definitive therapy (instead of empirical) as the results of urine culture were known before the admission (taken as outpatient). Of the remaining 285 cases included in the analysis of accuracy, the empirical therapy was accurate in 71.9%. When the empirical therapy was concordant with prior microbiological data, the rate of accuracy for treatment against the uropathogen improved from 7.3% to 92.7% (p<0.001). Importantly, all episodes (n = 190) that were given concordant therapy with previous culture were found to be accurate in the index episode (100%).

As shown in Table 3, the results of univariate analysis indicated that gender (p = 0.002) and reason of admission (p = 0.001) were significantly associated with the accuracy of treatment. In addition, the multivariate analysis identified both gender and reason of admission as independent factors that predicted the accuracy of treatment. Male cases were less accurately treated compared to female cases (OR = 0.528, 95CI% = 0.289–0.963, p = 0.037) (Table 3). Furthermore, antibiotic treatment in surgical patients was less accurate compared to cases treated in internal ward (OR = 0.451, 95CI% = 0.234–0.870, p = 0.018) (Table 3).

**Table 3:** Predictors of accurate empirical therapy of the index ESBL-UTI episode.

Predictor^a^	Univariate analysis			Multivariate analysis		
	Non accurate N = 80	Accurate N = 205	P value	OR	95%CI	P value
Age^b^	62 [47.5–73.75]	60 [29–72]	0.136	0.994	0.983–1.005	0.296
Gender			0.002			0.037
• Female	30 (37.5)	119 (58)		Ref.		
• Male	50 (62.5)	86 (42)		0.528	0.289–0.963	
Comorbidity			0.520			
• No	20 (25)	44 (21.5)				
• Yes	60 (75)	161 (78.5)				
Interval between paired isolates (months)^b^	3 [2–5]	3 [2–5]	0.673			
Reasons of admission			0.001			0.018
• Medical reasons	55 (68.8)	176 (85.9)		Ref.		
• Surgical reasons	25 (31.3)	29 (14.1)		0.451	0.234–0.870	
Presence of urological/surgical condition			0.084			0.839
• No	35 (43.8)	113 (55.1)		Ref.		
• Yes	45 (56.3)	92 (44.9)		0.939	0.511–1.725	
Negative Intervening culture			0.288			
• No	72 (90)	192 (93.7)				
• Yes	8 (10)	13 (6.3)				
Type of ESBL producer			0.591			
• *E*. *coli*	65 (81.3)	172 (83.9)				
• *K*. *pneumoniae*	15 (18.8)	33 (16.1)				

Abbreviations: OR, odds ratio; CI, confidence interval.

^a^ All data expressed as n (%) of participants unless otherwise indicated.

^b^ Data described as median [Interquartile range].

## Discussion

The results of our study demonstrate that postulation approach might be promising in clinical practice; prior positive ESBL cultures can allude to potential future similar episodes. Regarding current culture pathogenic identity, our study demonstrated that more than half of index urine cultures were ESBL-positive. This further highlights the potential usability of prior positive ESBL isolates to detect similar future isolates. Notably, our results replicated those reported by Schweizer et al who conducted a study in 2008 and reported the usefulness of prior colonization or infection with *methicillin-resistant Staphylococcus aureus* (MRSA) to predict the subsequent MRSA bacteremia [17]. In addition, a previous retrospective study by MacFadden et al, investigated the predictive utility of prior positive urine culture to determine identity and susceptibility of subsequent urine culture results, and yielded similar results [21].

In the present study, as for predictors of urine culture identity in index episodes, shorter monthly intervals between isolates in the defined episodes were predictive of accurate identification of ESBL in future episodes. Comparable results were reported by Dickestien et al who conducted a study in 2016 and found that higher odds of growing similar pathogens in future UTI episodes are associated with shorter interval between the episodes [22]. Additionally, our study indicated that the presence of surgical or urological conditions were associated with higher odds of developing future ESBL-UTIs. It is known that patients with comorbid conditions have frequent encounters at healthcare settings; lathering the way for ESBL colonization and recurrent infection [23]. It is assumed that vulnerability of patients with urological condition to acquire resistant bacterial strains is enhanced, as the host’s immune system is weakened [24–28]. Many previous studies showed that urinary instrumentation and/or a history of frequent UTIs were both linked to a higher hazard of ESBL-UTIs [25–28]. This finding is in line with current practice at the site of study conduction. At KAUH, clinicians assume that patients with urological conditions are at higher risk for UTI related complications. Hence, no risk is given to chance when dealing with potential ESBL-UTI episodes; the presence of a single previous ESBL documented urine culture, signifies an alarming clue to assume ESBL-induced UTI in any future occurrences of UTI episodes.

Early appropriate matching between the empiric antibiotic and the growing bacteria in index culture, as guided by the results of the previous positive culture, has the potential to reduce the rate of treatment failure and infection progression [29, 30]. Although many previous studies have investigated the susceptibility profiles in ESBL-UTIs [31–33], limited number of studies have evaluated approaches to enhance the accuracy of such therapy. Linsenmyer et al’s unique study assessed potential approaches to enhance empiric antibiotic selection accuracy in treating ESBL-UTIs [34]. In terms related to concordance and accuracy, the present study found that choosing empiric antibiotic therapy that was concordant with the preceding microbiologic data resulted in eradication in 92.7% of episodes. Only 7.3% of empiric antibiotic therapy options were accurate when the administered therapy was discordant from the earlier microbiologic results. The accuracy rate in treating the index UTI episodes was found to be 12.7 times greater than that documented when the utilized antibiotic agent was discordant with prior microbiological results. This rate of improved accuracy parallels the findings in the study conducted by Linsensmyer et al; the rate of accuracy for treatment has improved from 32% to 76% when empiric therapy was concordant with the prior microbiologic data [34].

In the present study, female gender was identified as an independent predictor for an improved rate in selecting accurate empirical therapy. Female gender is a well-known risk factor for UTIs in general, and for ESBL bacteria colonization and acquisition in particular [23]. The high-frequency of recurrent UTI in female gender was also reported [35]. Thus, it can be assumed this high-frequency among female has resulted in prototyping prior antibiotic use in this population either to treat potential sporadic UTI episodes or to treat recurrent episodes. The accuracy of empiric therapy of UTIs in females compared to males was not evaluated in previous studies to refute or accept such assumption.

Furthermore, hospitalization for medical reasons was reported as another independent predictor for an improved rate of accuracy. Most patients with urological conditions are admitted to medical ward at KAUH, the site of granted privilege to conduct our study. As aforementioned, a sole previous documented ESBL UTI episode raises clinician’s concern for potential similar incident and, hence justify the prescription of ESBL-resistant empiric antibiotics.

It is of paramount importance to note that the presence of a negative intervening culture between the two episodes has not affected the accuracy rate for the chosen antibiotic therapy. This finding reflects that the presence of ESBL-positive cultures in previous episodes has raised the suspicion of ESBL-positive cultures in any subsequent episode, even in the presence of an ESBL-negative intervening culture. This finding is parallel with that attained by Mac Fadden and his colleagues as susceptibility profile was the same or better in patients with negative intervening cultures [21].

UTIs are a good target for antibiotic resistance surveillance and stewardship programs, given the high incidence and the associated cost burden. Interestingly, novel resistance indicators and resistance classification terms have been investigated in UTIs. Older drug resistance terms, such as multidrug resistance (MDR) and pan drug resistance (PDR), might be substituted for more clinically-centered, reflective criteria. The new criteria, for example difficult-to-treat resistance (DTR) and modified DTR, relate resistance pattern against specific UTI antibiotics (such as fosfomycin, and trimethoprim-sulfamethoxazole) to clinical outcomes, such as making it more useful as a clinical aide in antibiotic selection [36].

To the best of our knowledge, our study was unique in being the first of its kind in the East Mediterranean Region (EMR) that examined and aided in validating the utility of previous microbiological results as an important simple rule to assist the clinician in treating suspected ESBL-UTIs. This stream of research is helpful for clinicians trying to balance antibiotic stewardship principles [e.g. avoiding unnecessary carbapenem use] with goal of achieving early adequate coverage. However, there are a few limitations to our study findings. Firstly, in retrospective studies, misclassification is a common concern but a clinical pharmacist in the current study had reviewed patients’ medical records for the purpose of validating exposure and outcome, rather than relying on diagnostic or billing codes. Secondly, there was no consistency in the selection of empiric antibiotics in our center, which might have impacted the clinical outcomes and study findings. Finally, our study was a single center study, and this can diminish the generalizability of the study results. A future research direction is to conduct prospective, multicenter studies to replicate our results in order to assess for potential addition as part of antibiotic stewardship program initiatives.

## Conclusions

Selecting an agent concordant with previous microbiologic data significantly increased the chance of accurate therapy for ESBL-UTIs. Clinician education is highly required to enhance the accuracy of empiric treatment of UTIs. Optimization of ESBL empiric antibiotic prescribing is a transdisciplinary approach; all members of healthcare team should be cognizant in dealing with potential ESBL-induced infections to balance the bar between both under and over prescription of broad-spectrum antibiotics. Improved ESBL treatment strategies is expected to limit cost burden on healthcare system, along with decreased mortality and morbidity rates and relieve associated pressure on hospital resources. Future prospective studies with a control arm might be helpful to confirm the results of present study. In addition, a research that focuses on test characteristics (sensitivity, specificity, positive predictive value, and negative predictive value) would be useful for the physicians to make reasonable decision about the utility of a prior urine culture.

## Supporting information

S1 File Microbiological lab data. (DOCX)

S2 File Demographic and clinical data. (DOCX)
