# Supplementary material for: Predictive utility of prior positive urine culture of extended- spectrum β -lactamase producing strains
Source: PLoS One. 2020 Dec 14;15(12):e0243741. doi: 10.1371/journal.pone.0243741 (PMC7735628; doi:10.1371/journal.pone.0243741)
Supplement: S1 File — (DOCX) [file pone.0243741.s001.docx]

**Microbiological data**

1. Urine culture results

Previous urine culture

- Date of the previous ESBL +ve urine culture (within 12 months of the current urine culture) ………………………..
- Type of microorganism in the previous urine culture…………………………..

Current urine culture:

- Date of current culture ......................................
- Results of current urine culture

1. Non ESBL producer (type of bacteria………………………………………..)
2. ESBL producer (type of bacteria………………………………………..)
3. Fungi
4. Others

1.mixed growth 2. no growth

- Culture taken before admission (outpatients cultures)

1. no
2. yes

- Negative intervening culture (between 2 consecutives positive ESBL cultures)

1. No
2. Yes
3. Sensitivity Data

Resistant (R) >> 0 Sensitive(S) >> 1 Intermediate(I)>> 2

| Current admission  Date: | Previous admission  Date: | Antibiotic |
| --- | --- | --- |
|  |  | ESBL |
|  |  | Ampicillin/sulbactam |
|  |  | ampicillin |
|  |  | Piperacillin tazobactam |
|  |  | piperacillin |
|  |  | Cefazolin |
|  |  | Ceftazidime |
|  |  | Ceftriaxone |
|  |  | Cefipime |
|  |  | Ertapenem |
|  |  | Imipenem |
|  |  | Meropenem |
|  |  | Amikacin |
|  |  | Gentamycin |
|  |  | Tobramycin |
|  |  | Ciprofloxacin |
|  |  | Levofloxacin |
|  |  | Nitrofurantoin |
|  |  | Trimeth/sulfa |
|  |  | Tigecycline |
|  |  | Colistin |
|  |  | cefuroxime |
|  |  | cefixime |
|  |  | Cefoxitin |
|  |  | aztreonam |
|  |  | moxifloxacin |
|  |  | minocycline |
|  |  | tetracycline |
|  |  | chloramphenicol |
|  |  | augmentin |
|  |  | nalidixic acid |
|  |  | norfloxacin |
|  |  | cefotaxime |
