# Supplementary material for: Predictive utility of prior positive urine culture of extended- spectrum β -lactamase producing strains
Source: PLoS One. 2020 Dec 14;15(12):e0243741. doi: 10.1371/journal.pone.0243741 (PMC7735628; doi:10.1371/journal.pone.0243741)
Supplement: S2 File — (DOCX) [file pone.0243741.s002.docx]

**Data collection sheet**

- Study ID………………………………
- Year of recruited cases ............................
- Age, (years)…………………………….
- Gender:

1. Female
2. Male

- Presence of comorbid condition

1. no
2. yes (specify……………………………………………………………..)

- Presence of surgical condition

1. no
2. yes (specify……………………………………………………………..)

- Presence of urologic conditions

1. no
2. yes (specify……………………………………………………………..)

- Antibiotics Allergy:

1. no
2. yes (specify type of antibiotic…………………………………………..)

- Reason of admission

1. medical reasons
2. surgical reasons

- Type of empirical therapy for treatment of current UTI……………………..………………
